# Evaluation of a Medicaid performance improvement project to reduce high-dose opioid prescriptions

**DOI:** 10.1186/s12913-022-07477-6

**Published:** 2022-01-14

**Authors:** Daniel M. Hartung, Jonah Geddes, Sara E. Hallvik, P. Todd Korthuis, Luke Middleton, Gillian Leichtling, Christi Hildebran, Hyunjee Kim

**Affiliations:** 1 Oregon State University, College of Pharmacy, 2730 S Moody Ave., CL5CP, Portland, OR 97201 USA; 2 Oregon Health & Science University, 3181 SW Sam Jackson Park Rc, Portland, Oregon 97239 USA; 3 Comagine Health, 650 NE Holladay St., Portland, OR 97232 USA

**Keywords:** Opioids, Medicaid, Overdose

## Abstract

**Background:**

In 2015, Oregon’s Medicaid program implemented a performance improvement project to reduce high-dose opioid prescribing across its 16 coordinated care organizations (CCOs). The objective of this study was to evaluate the effect of that program on prescription opioid use and outcomes.

**Methods:**

Using Medicaid claims data from 2014 to 2017, we conducted interrupted time-series analyses to examine changes in the prescription opioid use and overdose rates before (July 2014 to June 2015) and after (January 2016 to December 2017) implementation of Oregon’s high-dose policy initiative (July 2015 to December 2015). Prescribing outcomes were: 1) total opioid prescriptions 2) high-dose [> 90 morphine milligram equivalents per day] opioid prescriptions, and 3) proportion of opioid prescriptions that were high-dose. Opioid overdose outcomes included emergency department visits or hospitalizations that involved an opioid-related poisoning (total, heroin-involved, non-heroin involved). Analyses were performed at the state and CCO level.

**Results:**

There was an immediate reduction in high dose opioid prescriptions after the program was implemented (− 1.55 prescription per 1000 enrollee; 95% CI − 2.26 to − 0.84; *p* < 0.01). Program implementation was also associated with an immediate drop (− 1.29 percentage points; 95% CI − 1.94 to − 0.64 percentage points; *p* < 0.01) and trend reduction (− 0.23 percentage point per month; 95% CI − 0.33 to − 0.14 percentage points; *p* < 0.01) in the monthly proportion of high-dose opioid prescriptions. The trend in total, heroin-involved, and non-heroin overdose rates increased significantly following implementation of the program.

**Conclusions:**

Although Oregon’s high-dose opioid performance improvement project was associated with declines in high-dose opioid prescriptions, rates of opioid overdose did not decrease. Policy efforts to reduce opioid prescribing risks may not be sufficient to address the growing opioid crisis.

**Supplementary Information:**

The online version contains supplementary material available at 10.1186/s12913-022-07477-6.

## Background

Despite declines in opioid prescribing, opioid use remains several fold higher today relative to the early 1990s and prescription opioids are still involved in more than one-third of opioid-related deaths in the US [1, 2]. To confront this public health crisis, federal and state authorities have mounted diverse initiatives such as developing clinical practice guidelines that advocate safer opioid prescribing, implementing prescription drug monitoring programs, and advancing legislative or payer-based limits that encourage fewer and/or less risky opioid prescriptions [3]. However, the evidence that interventions and policies to reduce high-risk opioid prescribing are effective at reducing opioid-related overdoses has been mixed [4]. There are also concerns and growing evidence that rapid prescription opioid tapering or discontinuation may be associated with adverse outcomes such as increased use of illicit opioids, suicide, and other opioid-related harms [5–7].

Like the rest of the country, Oregon’s opioid-related overdose rate in 2017 was more than twice the rate observed in 2001 [8]. Efforts to address this public health crisis in Oregon have been diverse and included prior authorization for opioid prescriptions exceeding 120 morphine milligram equivalents (MME) that was limited to the fee-for-service program (~ 10% of Oregon Medicaid population), introduction of Oregon’s Prescription Drug Monitoring Program (PDMP) in 2011, and developing evidence-based reviews to support Medicaid coverage standards [9, 10]. Efforts to expand access to treatment for opioid use disorders during that time period, such as a federal grant to expand medication access in four rural counties that began in 2016, were generally small in scale. Similarly, naloxone access initiatives did not yet have wide reach.

In 2012, Oregon adopted an innovative approach to delivering care through its managed Medicaid program [11]. Oregon’s Medicaid program is administered through 16 Coordinated Care Organizations (CCOs), which are community-based healthcare delivery systems that coordinate physical, mental, addictions, and dental care and accept full financial risk for their members, similar to Accountable Care Organizations [11]. As part of this model, CCOs are required to conduct *performance improvement projects* that can improve care. CCO administrators collaboratively select the topics and outcome metrics for performance improvement projects. The first performance improvement project was directed at integration of physical and mental healthcare services and evaluated diabetes monitoring in people with serious mental illness [12]. The second statewide project involved prescription opioid safety. Specifically, in July 2015, Oregon’s Medicaid program introduced a performance improvement project to reduce high-dose opioid prescribing [12]. Specifically, Oregon’s performance improvement project aimed at decreasing the number of Medicaid enrollees filling prescriptions with > 90 daily morphine milligram equivalents [MME] and > 120 daily MME, and report back progress meeting those indicators in 2016 and 2017. Although CCOs were required to participate, the state granted CCOs flexibility to develop and implement programs and policies to meet their targets. In response, CCOs adopted various interventions including pharmacy benefit restriction (e.g. quantity limits and prior authorizations on long-acting opioids), provider directed interventions (e.g. training programs and targeted letters), and patient facing education [13].

The objective of this study was to examine trends in opioid prescribing and overdose-related outcomes under the Oregon CCO’s opioid performance improvement project that aimed to reduce high-dose opioid prescribing.

## Methods

### Data source and sample selection

We used Oregon Medicaid enrollment files, medical and pharmacy claims to identify patient demographics, health characteristics, prescription opioid use patterns, and rates of overdose. Our study period was July 2014 to December 2017, covering 12 months before and 30 months after the CCO performance improvement project started in July 2015.

The study sample included non-pregnant adults aged 18–64 with at least 1 month of continuous enrollment in an Oregon Medicaid CCO from 2014 to 2017. We excluded individuals with dual Medicare enrollment, those who resided in a long-term care facility, or had a cancer diagnosis code during the study period [14]. The effects of applying those criteria on the study sample are summarized in Supplemental Fig. 1.

### Outcomes

For each month during the study period, we calculated three prescription opioid use outcomes using pharmacy claims: 1) count of opioid prescriptions dispensed per 1000 enrollees, 2) count of high-dose opioid prescriptions per 1000 enrollees, 3) proportion of opioid prescriptions dispensed with a high-dose. Prescription opioids were identified using National Drug Code identifiers derived from FirstDataBank’s Drug File. Consistent with CDC Guideline for Prescribing Opioids for Chronic Pain and the threshold selected for most CCOs, we defined high dose as any prescription exceeding 90 MME per day [13, 15]. We calculated daily MME for each prescription by multiplying the formulation strength by the quantity dispensed and the CDC endorsed conversion factor [16] and then dividing by each prescription’s day supply.

We measured rates of opioid overdose using medical claims. We defined opioid overdoses as opioid-related emergency department (ED) encounters or inpatient admissions using International Classification of Disease (ICD) 9th and 10th Revision codes that indicated opioid poisoning as described in Supplemental Table 1. ED encounters were identified in Medicaid medical claims using revenue center codes (450–459, 0981). We calculated the number of opioid overdoses overall and separately based on if heroin was involved (ICD9 96,501, E8500; ICD10 T401). Thus, we delineated three opioid overdose outcomes: 1) total opioid overdoses per 100,000 enrollees, 2) heroin-involved overdoses per 100,000 enrollees, and 3) non-heroin involved overdoses per 100,000 enrollees.

### Statistical analysis

We conducted interrupted time series (ITS) regressions to evaluate changes in opioid prescribing and overdose outcomes before and after Oregon’s CCO performance improvement project overall and separately for each CCO. Because the initiative was implemented gradually and inconsistently by CCOs, we omitted the first 6 months of the intervention (July 2015 to December 2015) as a transition period. Thus, we ultimately had 12 months of observation before the intervention (July 2014 to June 2015) and 24 months of observation after the intervention (January 2016 to December 2017).

We used two sets of ITS regressions. First, we included all 16 CCOs in the regression and evaluated the overall association of the program with outcomes. Second, we stratified the sample by each CCO and estimated regressions for each CCO to evaluate heterogeneity across the CCOs. In both sets of regressions, the unit of analysis was CCO-month.

The general form of our ITS regression model was Y = β_0_ + β_1_*PreTrend + β_2_*PIP + β_3_*PostTrend + εt, where Y is the dependent variable of interest, PreTrend continuous variable to indicate month (1 to 36), PIP indicates if the observation was before or after Oregon’s performance improvement project was implemented, and PostTrend is a counting variable to indicate the month after implementation (0 if before and 1 to 24 after). β_1_ estimates the trend before implementation, β_2_ is immediate change following implementation, and β_3_ is the change in trend following program implementation. We estimated all ITS regressions based on ordinal least squares with Newey-West standard errors to account for heteroskedasticity. We adjusted for first-degree autocorrelation and we considered 2-tailed *P* < 0.05 to be statistically significant.

A secondary objective was to determine if CCO reductions in high-dose opioid prescribing were associated with reduced opioid overdose. Because overdose events are relatively uncommon and most CCOs enrolled fewer than 100,000 individuals, we grouped CCOs into two groups according to if they experienced a significant reduction in the trend in the proportion of opioid prescriptions over 90 MME per day following the performance improvement project. We then analyzed trends in heroin-involved, non-heroin involved, and total opioid overdose within these two CCO groupings.

We performed all analyses using Stata, version 16 (StataCorp) and conducted our ITS analysis through the user written “itsa” command [17]. The study protocol was performed in accordance with the relevant human subjects guidelines. This study was approved by the institutional review board at the Oregon Health & Science University. Because this was a retrospective analysis of de-identified data with minimal risks, a waiver of informed consent was granted.

## Results

The final dataset included 584,720 adults across 16 different CCOs. Table 1 summarizes demographic and opioid use characteristics for these individuals. Patient demographics across CCOs were similar in enrollee age and sex, but differed by rural/urban designation, prescription opioid use, high-dose prescription opioid use, and opioid overdose rates. The largest CCOs (Health Share and Family Care) were operating in the Portland Metro region and had 8 and 11% of their enrollees residing in a rural location, respectively. In contrast, many of the smaller CCOs predominately comprised enrollees living in a rural area. Overall, 9% prescription opioid users had high dose prescriptions (≥ 90 daily MME), but the prevalence varied more than 3-fold across CCOs, ranging from 4 to 14%.

**Table 1:** Enrollee characteristics and prescription opioid use across 16 Coordinated Care Organizations (CCOs) during the pre-intervention period

CCO	Enrollees	% Female	% Rural residence	Mean age (sd)	% of enrollees with any Rx opioid*	% of enrollees with an opioid Rx who had an Rx ≥90 MME per day*	Opioid overdoses per 100,000*
**Portland Metro**							
Health Share of Oregon	135,527	48	8	36 (11)	10.9	9.2	21
Family Care	99,169	46	11	36 (11)	6.7	7.3	20
Columbia Pacific	18,112	49	93	38 (12)	15.4	12.2	10
**Mid Valley**							
Yamhill Community Care	15,015	49	89	37 (12)	11.0	7.2	4
Willamette Valley Community Health	50,025	48	28	36 (12)	9.6	8.8	6
Intercommunity Health Network	36,080	48	50	36 (12)	13.9	9.2	7
Trillium Community Health Plan	60,374	48	26	36 (12)	13.6	10.6	13
**Central / Eastern Oregon**							
PacificSource Community Solutions CCO Central Oregon	35,175	48	55	37 (12)	11.8	4.3	9
PacificSource Community Solutions CCO Columbia Gorge	7502	48	96	38 (12)	9.0	6.3	11
Eastern Oregon CCO	28,133	49	97	37 (12)	15.4	9.6	6
**Southern Oregon**							
Cascade Health Alliance	11,088	49	95	37 (12)	7.8	3.8	2
Umpqua Health Alliance	17,175	47	95	37 (12)	6.3	7.9	12
Western Oregon Advanced Health	13,854	48	96	38 (12)	5.9	5.4	2
AllCare Health Plan	31,135	46	58	37 (12)	5.7	7.1	12
Jackson Care Connect	17,722	48	28	36 (12)	13.4	14.2	15
PrimaryHealth	8634	44	94	37 (12)	6.9	8.8	6
**Total**	584,720	47.5	38.3	39 (13)	10.4	8.9	10

*CCO* Coordinated care organization, *MME* Morphine milligram equivalent, *sd* Standard deviation, *Rx* Prescription

*average monthly rate per 100,000 during baseline period

**Fig. 1:**
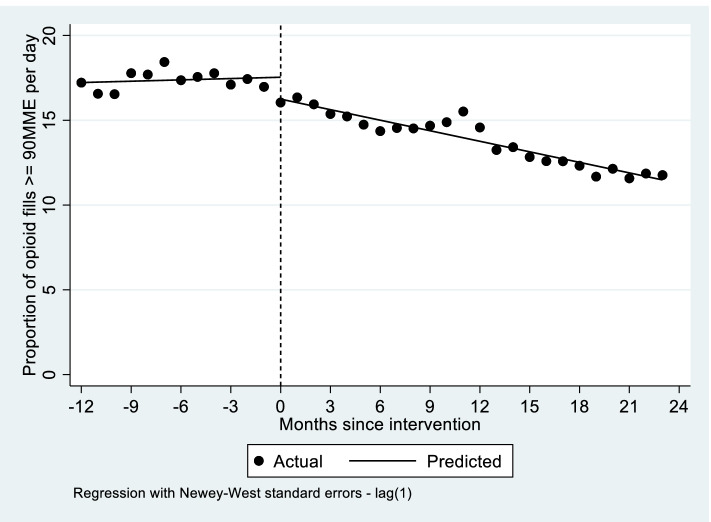
Interrupted time series regression model of proportion of opioid fills > = 90 MME per day with fitted trend lines before and during performance improvement project. Notes: Dotted line indicates start of performance improvement project

Table 2 summarizes estimates from the three ITS regression models. As shown in Fig. 1, the proportion of all opioid prescriptions that were > =90 MME declined immediately by 1.29 percentage points (95% CI − 1.94 to − 0.64 percentage points; *p* < 0.01). There was also a significant decline in the monthly trend of 0.23 percentage points per month (95% CI − 0.33 to − 0.14 percentage points; *p* < 0.01). Trends in total opioid and high-dose opioid prescriptions are shown in Supplemental Fig. 2. The trend in total opioids prescription was declining prior to the CCO initiative at a rate of − 2.13 prescriptions per 1000 enrollees per month (95% CI − 2.61 to − 1.65; *p* < 0.01). Following the implementation of the CCO initiative, the trend moderated significantly but remained negative. There was an immediate reduction in high dose opioid prescriptions after the CCO initiative (− 1.55 prescription per 1000 enrollee; 95% CI − 2.26 to − 0.84; *p* < 0.01), but no significant change in the overall monthly trend.

**Table 2:** Results from interrupted time series regressions of opioid utilization and opioid overdose outcomes before and during performance improvement project

	Opioid prescriptions per 1000 enrollees per month			High-dose opioid prescriptions per 1000 enrollees per month			Proportion of opioid prescriptions that are high dose		
	Coefficient	95% CI	*p*-value	Coefficient	95% CI	p-value	Coefficient	95% CI	*p*-value
Intercept	115.89	112.37 to 119.41	< 0.01	19.94	19.16 to 20.72	< 0.01	17.22	16.51 to 17.94	< 0.01
Baseline trend	−2.13	−2.61 to −1.65	< 0.01	−0.34	−0.43 to −0.25	< 0.01	0.03	−0.06 to 0.12	0.56
Immediate change	−1.07	−4.85 to 2.71	0.57	−1.55	− 2.26 to − 0.84	< 0.01	−1.29	− 1.94 to − 0.64	< 0.01
Trend change	1.29	0.80 to 1.79	< 0.01	0.06	−0.04 to 0.15	0.23	−0.23	− 0.33 to − 0.14	< 0.01

*CI* Confidence interval

**Fig. 2:**
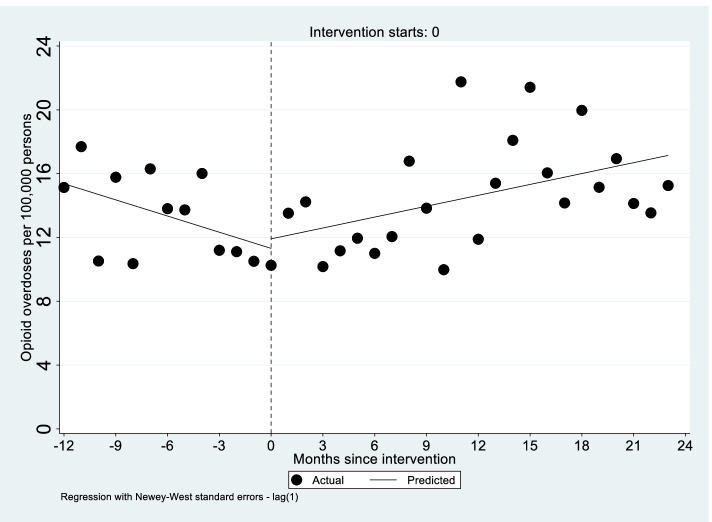
Interrupted time series regression models of opioid overdose per 100,000 per month with fitted trend lines before and during performance improvement project. Notes: Dotted line indicates start of performance improvement project

Trends in total opioid overdose are presented in Fig. 2 with corresponding ITS regression estimates summarized in Table 3. In the 12 months prior to the CCO initiative, trends in opioid overdose were decreasing significantly. After implementation of the CCO initiative, there was a significant increase in total opioid overdoses (0.57 overdoses per 100,000 enrollees per month; 95% CI 0.26 to 0.87; *p* < 0.01). As illustrated in Supplemental Fig. 3, increases in the overdose trend were significant for both heroin-involved (0.24 overdoses per 100,000 enrollees per month; 95% CI 0.01 to 0.47; *p* = 0.04) and non-heroin involved overdoses (0.40 overdoses per 100,000 enrollees per month; 95% CI 0.16 to 0.64;;< 0.01). For each outcome, the increase was large enough to reverse the previously declining overdose rates.

**Table 3:** Results from interrupted time series regressions of opioid overdose outcomes before and during performance improvement project overall and by Coordinated Care Organizations (CCOs) high dose prescribing changes

	Any Opioid Overdose			Heroin-involved			Non-heroin involved		
	Coefficient	95% CI	*p*-value	Coefficient	95% CI	*p*-value	Coefficient	95% CI	*p*-value
**All CCOs**									
Intercept	15.37	13.71 to 17.03	< 0.01	8.69	7.93 to 9.45	< 0.01	7.26	5.42 to 9.10	< 0.01
Pre trend	−0.34	−0.60 to −0.08	0.01	−0.08	− 0.26 to 0.09	0.34	− 0.3	− 0.52 to − 0.07	0.01
Immediate change	0.60	−2.04 to 3.23	0.65	−1.18	−3.03 to 0.67	0.20	1.9	0.10 to 3.69	0.04
Trend change	0.57	0.26 to 0.87	< 0.01	0.24	0.01 to 0.47	0.04	0.40	0.16 to 0.64	< 0.01
**10 CCOs with a significant decrease in proportion of opioid fills > =90 MME per day**									
Intercept	17.81	15.97 to 19.65	< 0.01	11.03	9.62 to 12.44	< 0.01	7.53	5.43 to 9.62	< 0.01
Pre trend	−0.40	−0.71 to −0.1	0.01	−0.11	− 0.37 to 0.16	0.43	− 0.37	− 0.62 to − 0.12	0.01
Immediate change	0.25	−2.83 to 3.32	0.87	−2.27	−4.93 to 0.38	0.09	2.74	0.81 to 4.67	0.01
Trend change	0.67	0.3 to 1.03	< 0.01	0.30	−0.04 to 0.63	0.08	0.48	0.2 to 0.75	< 0.01
**6 CCOs without a significant decrease in proportion of opioid fills > =90 MME per day**									
Intercept	9.92	7.04 to 12.79	< 0.01	3.45	2.43 to 4.48	< 0.01	6.66	3.89 to 9.43	< 0.01
Pre trend	−0.17	−0.63 to 0.29	0.46	−0.01	−0.14 to 0.12	0.88	−0.15	− 0.59 to 0.29	0.49
Immediate change	1.31	−3.43 to 6.04	0.58	1.09	−0.58 to 2.76	0.19	0.13	−3.74 to 4.01	0.94
Trend change	0.33	−0.19 to 0.86	0.20	0.10	−0.1 to 0.3	0.31	0.24	−0.22 to 0.7	0.30

*MME* Morphine milligram equivalents, *CCO* coordinated care organization, *CI* confidence interval

Supplemental Table 2 summarizes ITS regression estimates for each CCO for the proportion of opioid prescriptions above 90 MME per day. The trend in percentage of opioid prescriptions over 90 MME per day declined significantly in 10 of 16 CCOs. Table 2 also summarizes ITS regression models for opioid overdose for the ten CCOs that had significant decline in the trend of opioid prescriptions over 90 MME per day and six that did not. Among the 10 CCOs that had reductions in high-dose opioid prescriptions, the trend in opioid overdose increased significantly (0.67 events per 100,000 enrollees per month; 95% CI 0.3 to 1.03; *p* < 0.01). These changes were largely driven by the significant increase in non-heroin involved overdose trends (0.48 events per 100,000 enrollees per month; 95% CI 0.2 to 0.75; *p* < 0.01). Although overdose rates also increased among the 6 CCOs that did not have reductions in their high dose opioid use, these changes were not statistically significant. Changes in heroin-involved overdose were not statistically significant when stratified by CCO groups.

## Discussion

States, healthcare systems, and payers have mounted diverse responses to the opioid epidemic. A common approach has been restrictions on opioid prescribing through development and adoption of prescription drug monitoring programs, clinical practice guidelines, and legal and payer strategies to limit certain types of opioid prescribing. However, the evidence that supply-side restrictions on opioid prescribing are effective at reducing opioid overdose has been mixed [3, 4].

In 2015, Oregon implemented an innovative performance improvement project across its 16 CCOs aimed at reducing high-dose opioid prescribing. Similar to national trends [18], overall and high-dose opioid prescribing prior to the implementation of the project were already in decline. Following introduction of the state’s high-dose initiative, we found the trajectory of high-dose opioid prescriptions, as a proportion of all opioid prescriptions, declined significantly. This reduction was a combination of an immediate reduction and continued decline in high-dose prescriptions observed following implementation of the state’s performance improvement project. Despite lower use of high-dose opioid prescriptions, we found no evidence the project was associated with reductions in opioid-related overdose hospitalizations. In contrast, there were significant increases in the trend for total, heroin-involved, and non-heroin involved opioid poisonings following the project’s implementation. Increases in opioid-related overdose trends were most pronounced among the ten CCOs that exhibited significant reductions in the proportion of high-dose opioid prescriptions.

Like the Oregon Medicaid policy initiative, many states have adopted guidelines or enacted laws that place explicit limits on opioid prescriptions [19]. Although evaluations of these initiatives have generally shown them to be effective at reducing different aspects of prescription opioid use, their effect on health outcomes is unclear. In 2007, the state of Washington became the first governmental authority to develop and implement opioid dosing guidelines that specifically cautioned against prescribing opioids over a specific dose threshold (120 MME per day). Several studies have found that implementation of the original guideline and subsequent iterations was associated with fewer high-dose prescriptions in the workers compensation and Medicaid programs [20–22]. Another state-level ecologic study found that states that implemented dose-related opioid prescribing guidelines (including Washington) had significant reductions for trends in hospitalization for opioid overdose compared to comparison states [23].

Public and commercial healthcare payers have also introduced polices such as prior authorization and quantity limits that are intended to limit opioid prescriptions [3, 4, 24–26]. In general, utilization management policies directed at opioid prescribing have been shown to have potent effects on opioid prescribing [27–31]. However, the effect of these payer strategies on opioid-related outcomes has also been mixed. Restrictions on high-dose and long-acting opioid use in two state Medicaid programs was associated with fewer high-risk opioid prescriptions, but was not associated with fewer opioid overdoses [30, 31]. However, another study found that Medicaid plans with more prior authorization policies had lower rates of opioid overdose [32].

In addition to the lack of strong evidence supporting policies restricting prescription opioid use on health outcomes, there have been growing concerns that supply side efforts to reduce opioid misuse, addiction, and overdose are challenging for patients with chronic pain, dependence, or untreated opioid use disorder if they are discontinued from opioid therapy. Qualitative research conducted among individuals with chronic pain indicate that prescription policies aimed at reducing opioid prescribing can have negative effects on patient stigmatization and fears of untreated pain [33, 34]. In a qualitative study involving individuals with a prior prescription opioid overdose, Mueller et al. found patients with chronic pain who experienced opioid-related policy restrictions sometimes engaged in higher risk behavior in attempts to manage pain exacerbations that included not taking pain medications as prescribed, co-administering with other substances such as alcohol, or illicitly obtaining opioids [34]. A growing number of quantitative studies have begun to examine the effect of restrictive opioid policies on potential unintended outcomes such as transition to illicit opioids, overdose, or self-harm. In a large cohort of prescription opioid users in the Veterans Health Administration reported that individuals who discontinued opioid therapy were at a higher risk of death from overdose or suicide relative to individuals who continued opioid therapy [5]. Another study in the Vermont Medicaid program found rapidity of dose reduction was associated with an increased risk of opioid-related adverse events among those who were discontinued from chronic opioid therapy [6]. A case-control study from a large integrated health care network found a two-fold higher odds of prescription opioid discontinuation among individuals who used heroin relative to non-heroin users [7].

Since the study period, Oregon has increased efforts to expand access to substance use disorder treatment, recovery support, and harm reduction tools, including through securing federal grants, investing state resources, and implementing policy changes. Our study suggests prescription opioid policy restrictions are insufficient in achieving reductions in overall overdose rates, and investment in a broader array of supports related to opioid use disorder is needed. Evidence has also grown supporting the use of buprenorphine therapy in opioid tapering protocols among patients without a diagnosis of opioid use disorder [35, 36].

This study has limitations. First, our study did not include a control group and therefore that observed reductions may have been affected by other secular trends in prescribing. Another methodologic limitation concerns the transition from ICD9 to ICD10 that occurred in October 2015, about the same time as the CCO initiative. However, in a national study hospitalization data admissions for poisonings actually declined following the transition which suggests the increases we observed may be conservative [37]. Our analyses were confined to Medicaid pharmacy claims data and did not capture opioid prescriptions that were paid for out-of-pocket [38]. It is possible that efforts to reduce high dose prescriptions were circumvented by patients paying cash for prescriptions where policy barriers were meant to restrict access.

It is also conceivable the rising overdose ED visits and hospitalizations were driven by fentanyl involvement. Illicit fentanyl has been the major contributor to rising opioid-related deaths over the last several years [39]. Nationally, deaths attributed to synthetic opioids, such as fentanyl, have quadrupled from 3.1 to 11.4 deaths per 100,000 from 2015 to 2019 [40]. Although, until recently, much of this rise has been concentrated in communities east of the Mississippi River, western parts of the US are now experiencing surges in fentanyl-involved deaths [41]. According to data from Oregon State Medical Examiner, fentanyl-related fatalities have increased three-fold from 0.399 to 1.464 deaths per 100,000 between 2015 and 2019 [42]. Fentanyl poisoning is normally coded in ICD10 as T40.4 “Poisoning by synthetic narcotics.” Although the validity of administrative data to identify opioid overdoses has been established, the specificity of ICD-10 data to correctly identify overdoses attributable to illicit fentanyl is unclear [43]. We attempted to separately analyze opioid-involved overdoses involving synthetics (ICD10 T40.4) and found they only accounted for about 5% of total opioid-related overdoses since October 2015. Finally, we did not examine other clinical consequences of policies which limit opioid supply such as inadequate pain control, suicide/suicidal ideation, and use of illicit drugs.

## Conclusions

Efforts to confront the opioid epidemic have often focused on reducing risks associated with prescription opioids, such as high-dose prescribing. We found that implementation of a quality improvement program aimed at reducing high-dose opioid prescribing across Oregon’s CCOs was associated with declines in these high-risk prescriptions. While rates of opioid overdose increased following the program’s implementation, the causal link between these two disparate trends remains murky because of the concurrent rise of fentanyl-involved poisonings in the West, which may not be fully delineated in administrative claims [44]. It is also possible that the intent of the policy was blunted by rising out-of-pocket payments for opioids, which are relatively low cost. With growing concerns about potential substitution of prescription opioids with those from illicit sources, our findings further underscore the limitations of supply-side efforts to combat a public health crisis that has evolved beyond prescription opioids.

## Supplementary Information

**Additional file 1.**

## Data Availability

The datasets generated and/or analyzed during the current study are not publicly available because of terms of the data use agreement.

## Abbreviations

CCO: Coordinated Care Organization
ED: Emergency department
ICD: International Classification of Disease
ITS: Interrupted Time Series
MME: Morphine Milligram Equivalent
