# Supplementary material for: Evaluation of a Medicaid performance improvement project to reduce high-dose opioid prescriptions
Source: BMC Health Serv Res. 2022 Jan 14;22:68. doi: 10.1186/s12913-022-07477-6 (PMC8760718; doi:10.1186/s12913-022-07477-6)
Supplement: Supplementary file 1 — Additional file 1. [file 12913_2022_7477_MOESM1_ESM.docx]

Supplemental Table 1: International Classification of Disease 9^th^ and 10^th^ Revision codes for opioid-related poisoning.

| **Code** | **Description** | **ICD-9-CM Code** | **ICD-10-CM Code** |
| --- | --- | --- | --- |
| 96500 | Poisoning by opium (alkaloids), unspecified | X |  |
| 96501 | Poisoning by heroin | X |  |
| 96502 | Poisoning by methadone | X |  |
| 96509 | Poisoning by other opiates and related narcotics | X |  |
| E8500 | Accidental poisoning by heroin | X |  |
| E8501 | Accidental poisoning by methadone | X |  |
| E8502 | Accidental poisoning by other opiates and related narcotics | X |  |
| T400x | Poisoning by opium |  | X |
| T401x | Poisoning by heroin |  | X |
| T402x | Poisoning by other opioids |  | X |
| T403x | Poisoning by methadone |  | X |
| T404x | Poisoning by synthetic narcotics |  | X |

Supplemental Figure 1: Flow diagram of effect of inclusion and exclusion criteria on study sample.

Cumulative Medicaid enrollment, January 2014 to December 2017: 1,775,262

966,432

Ages <18 or >64: 808,830

Dual eligibles: 78,850

887,582

Pregnant women: 99,264

Cancer diagnosis: 85,503

<1 month enrollment: 105,798

788,318

702,815

690,518

584,720

Long-term care: 12,297

Supplemental Figure 2: Interrupted time series regression models of a) prescription opioid fills per 1000 enrollees per month b) prescription opioid fills +90 MME a day per 1000 enrollees per month.

Notes: Dotted line indicates start of performance improvement project.

Supplemental Figure 3: Interrupted time series regression model of a) heroin-involved opioid overdose per 100,000 per month b) non-heroin involved opioid overdose per 100,000 per month.

Notes: Dotted line indicates start of performance improvement project.

Supplemental Table 2: Interrupted time series regression models of proportion of opioid fills with >=90 morphine milligram equivalents (MME) per day for all Coordinated Care Organizations (CCOs).

| CCO Name | Coefficient | 95% CI | | p |
| --- | --- | --- | --- | --- |
| **CCOs without a significant decline in trend** | | | | |
| Western Oregon Advanced Health | | | | |
| Intercept | 7.98 | 4.80 | 11.15 | <0.01 |
| Pre Trend | 0.09 | -0.34 | 0.53 | 0.67 |
| Level Change | 0.98 | -2.35 | 4.32 | 0.55 |
| Trend Change | -0.20 | -0.68 | 0.28 | 0.40 |
| AllCare Health Plan |  |  |  |  |
| Intercept | 12.72 | 11.87 | 13.56 | <0.01 |
| Pre Trend | -0.01 | -0.19 | 0.16 | 0.87 |
| Level Change | -2.97 | -5.31 | -0.63 | 0.01 |
| Trend Change | -0.11 | -0.31 | 0.09 | 0.26 |
| Cascade Health Alliance | | | | |
| Intercept | 8.77 | 6.70 | 10.84 | <0.01 |
| Pre Trend | -0.31 | -0.56 | -0.05 | 0.02 |
| Level Change | 4.37 | 2.36 | 6.38 | <0.01 |
| Trend Change | -0.05 | -0.33 | 0.24 | 0.74 |
| Columbia Pacific |  |  |  |  |
| Intercept | 24.26 | 22.38 | 26.15 | <0.01 |
| Pre Trend | -0.10 | -0.38 | 0.19 | 0.50 |
| Level Change | -0.39 | -2.69 | 1.91 | 0.73 |
| Trend Change | -0.29 | -0.60 | 0.01 | 0.06 |
| Trillium Community Health Plan | | | | |
| Intercept | 20.39 | 19.34 | 21.44 | <0.01 |
| Pre Trend | -0.04 | -0.17 | 0.10 | 0.57 |
| Level Change | -0.29 | -1.35 | 0.78 | 0.59 |
| Trend Change | -0.04 | -0.19 | 0.10 | 0.54 |
| Willamette Valley Community Health | | | | |
| Intercept | 17.75 | 17.29 | 18.21 | <0.01 |
| Pre Trend | -0.32 | -0.44 | -0.20 | <0.01 |
| Level Change | -2.10 | -3.61 | -0.59 | 0.01 |
| Trend Change | 0.16 | 0.02 | 0.31 | 0.03 |
| **CCOs with a significant decline in trend** | | | | |
| Eastern Oregon CCO | | | | |
| Intercept | 18.82 | 16.95 | 20.68 | <0.01 |
| Pre Trend | 0.06 | -0.19 | 0.31 | 0.61 |
| Level Change | 0.21 | -1.25 | 1.68 | 0.77 |
| Trend Change | -0.3 | -0.56 | -0.05 | 0.02 |
| Family Care |  |  |  |  |
| Intercept | 12.71 | 11.91 | 13.51 | <0.01 |
| Pre Trend | 0.07 | -0.02 | 0.17 | 0.11 |
| Level Change | 1.07 | -0.12 | 2.27 | 0.08 |
| Trend Change | -0.16 | -0.28 | -0.04 | 0.01 |
| Health Share of Oregon | | | | |
| Intercept | 18.72 | 17.65 | 19.79 | <0.01 |
| Pre Trend | 0.16 | 0.03 | 0.29 | 0.02 |
| Level Change | -2.81 | -3.71 | -1.91 | <0.01 |
| Trend Change | -0.36 | -0.49 | -0.23 | <0.01 |
| Intercommunity Health Network | | | | |
| Intercept | 19.27 | 18.49 | 20.06 | <0.01 |
| Pre Trend | -0.23 | -0.33 | -0.12 | <0.01 |
| Level Change | -0.43 | -2.01 | 1.15 | 0.58 |
| Trend Change | -0.25 | -0.39 | -0.11 | <0.01 |
| Jackson Care Connect | | | | |
| Intercept | 27.21 | 26.16 | 28.26 | <0.01 |
| Pre Trend | -0.06 | -0.17 | 0.05 | 0.27 |
| Level Change | -5.54 | -7.02 | -4.07 | <0.01 |
| Trend Change | -0.37 | -0.51 | -0.23 | <0.01 |
| Pacific Source |  |  |  |  |
| Intercept | 7.41 | 6.28 | 8.54 | <0.01 |
| Pre Trend | 0.29 | 0.08 | 0.49 | 0.01 |
| Level Change | -1.42 | -3.21 | 0.38 | 0.12 |
| Trend Change | -0.38 | -0.58 | -0.18 | <0.01 |
| Pacific Source Gorge | | | | |
| Intercept | 10.76 | 9.27 | 12.24 | <0.01 |
| Pre Trend | 0.17 | -0.02 | 0.35 | 0.07 |
| Level Change | 0.21 | -2.54 | 2.96 | 0.88 |
| Trend Change | -0.51 | -0.75 | -0.26 | <0.01 |
| PrimaryHealth |  |  |  |  |
| Intercept | 15.09 | 11.67 | 18.50 | <0.01 |
| Pre Trend | 0.08 | -0.50 | 0.66 | 0.78 |
| Level Change | -0.42 | -5.38 | 4.53 | 0.86 |
| Trend Change | -0.68 | -1.31 | -0.06 | 0.03 |
| Umpqua Health Alliance | | | | |
| Intercept | 9.85 | 7.86 | 11.83 | <0.01 |
| Pre Trend | 0.32 | 0.06 | 0.58 | 0.02 |
| Level Change | -1.27 | -3.26 | 0.72 | 0.20 |
| Trend Change | -0.66 | -0.96 | -0.36 | <0.01 |
| Yamhill Community Care | | | | |
| Intercept | 13.67 | 12.59 | 14.75 | <0.01 |
| Pre Trend | 0.10 | -0.05 | 0.26 | 0.19 |
| Level Change | -0.70 | -2.21 | 0.81 | 0.35 |
| Trend Change | -0.27 | -0.45 | -0.09 | <0.01 |
